# Efficacy of a Web-Based Executive Function Training Program to Induce Healthier Food Choices and Increase Valuation of Fruits and Vegetables in Adults: Protocol for a Randomized Controlled Trial

**DOI:** 10.2196/66394

**Published:** 2025-09-16

**Authors:** James N Roemmich, Alese M Nelson, Eric Stice, Julie M Hess, Daniel G Palmer, Shanon L Casperson

**Affiliations:** 1 Grand Forks Human Nutrition Research Center, United States Department of Agriculture's Agricultural Research Service Grand Forks, ND United States; 2 Department of Psychology, University of Wisconsin Oshkosh Oshkosh, WI United States; 3 Department of Psychiatry and Behavioral Sciences, Stanford University Stanford, CA United States

**Keywords:** executive function, vegetables, fruits, diet, food choices, energy-dense foods, nutrient-poor foods, nutrient-dense foods

## Abstract

**Background:**

Executive function (EF) is a set of explicit (top-down) cognitive abilities theorized to assist in conscious control of eating behavior. However, it is unclear whether EF training can improve valuation and consumption of nutrient-dense foods while concurrently altering attitudes toward and perceptions of nutrient-poor foods and their consumption.

**Objective:**

The primary objective is to determine the efficacy of a web-based EF training program designed to concurrently improve attitudes toward and perceptions and consumption of nutrient-dense foods (fruits and vegetables) while worsening those of nutrient-poor foods (ie, candy and fried snacks).

**Methods:**

Participants include a nationally representative US sample (N=500) of men and women aged 18 to 70 years with a BMI between 18.5 and 38 kg/m^2^. Potential participants are recruited through an open link to a prescreening questionnaire. Qualified participants are sent an electronic version of the informed consent form to sign. After consenting, they are invited to create an account on our semiautomated web-based platform and randomized to EF training via a suite of 4 web-based games that promote responding to fruits and vegetables, inhibit responses to nutrient-poor foods, and train attention toward the former and away from the latter or sham training that involves images of flowers and songbirds (control). Training occurs at least twice per week. EF is tested before and after 8 weeks of training. Dietary intake is measured before and after the 8-week intervention. Group and sex differences in frequency of participants violating inhibitory signals will be assessed using log-linear models. Linear mixed-effects models will test whether web-based EF training improves EF as measured via response inhibition and attention during computer-based gameplay. The independent and interactive associations between inhibitory and attentional learning and consumption of nutrient-poor foods will be tested using mixed model regression. If random assignment results in baseline imbalances across conditions on any variables, they will be used as covariates in the mixed models. We will use both an intention-to-treat analysis using (restricted) maximum likelihood estimation and a completer analysis to understand the results from those participants who completed the training at least twice per week.

**Results:**

As of June 1, 2025, overall, 899 individuals have applied to participate in the study. Of 899 applicants, 763 (84.9%) qualified and 630 (70.1%) consented. There have been 386 withdrawals due to noncompliance with study requirements (n=342, 88.6%) or identification as a bot (n=35, 9.1%).

**Conclusions:**

The association between improved EF and healthy eating behaviors may be key to improving diet quality. If effective, this web-based EF training program will provide a platform that can be made widely available to aid individuals in food-related decision-making. The platform could be modified to target other food choices and other health behaviors, such as reducing sedentariness and increasing physical activity.

**Trial Registration:**

ClinicalTrials.gov NCT05938894; https://clinicaltrials.gov/study/NCT05938894

**International Registered Report Identifier (IRRID):**

DERR1-10.2196/66394

## Introduction

### Background

There is a correlation between healthy behaviors and constructs of executive function (EF) [1]. Therefore, the purpose of this study is to determine whether a web-based EF training program can improve response inhibition and attention and concurrently improve attitudes toward and perceptions and consumption of nutrient-dense foods (ie, fruits and vegetables [FV]) while worsening those of nutrient-poor foods (ie, candy and fried snacks). In effect, the aim is to test whether a web-based EF training program can promote the substitution of nutrient-poor foods with FV. Greater FV consumption is associated with better weight control; reduced risk of all-cause mortality, cardiovascular disease, diabetes, and cancer; and better eye and skin health [2,3]. There is also an association with better mental and cognitive health [4-6]. Thus, EF training that effectively promotes increased FV intake will benefit both physical and mental health. It remains unclear whether EF training can be successfully implemented entirely remotely and independent of behavioral cognitive training.

### EF Definition

EF is a set of explicit (top-down) cognitive abilities theorized to assist in conscious control of behavior, for instance, the ability to override impulsive (implicit) processes. Collectively, these processes exert influence on the decisions people make that drive appetitive behaviors; poorer EF is associated with unhealthy dietary choices [7,8]. Constructs of EF that are of particular interest and used in this project include (1) inhibitory motor control, or the ability to override impulsive responses; and (2) attentional control, or attention bias toward food cues in the environment.

Individuals who display personality traits associated with impulsiveness and inhibitory control deficits or those who show less recruitment of inhibitory regions of the brain are more sensitive to food cues and may be more vulnerable to the pervasive presence of food, particularly highly palatable nutrient-poor foods [9-11]. These individuals have a preference for immediate food reward [12,13] and poorer weight control [10,14,15]. Greater attention bias toward food cues predicts greater increases in body weight [16]. Food-related attention bias positively correlates with food craving, food intake, and hunger [17], and this may be more pronounced in individuals with overweight and obesity [11,18-21]. Furthermore, individuals who exhibit greater neural activation to energy-dense food images in reward and attention regions of the brain (eg, the orbitofrontal cortex and caudate nucleus) are more sensitive to food cues and more vulnerable to the pervasive temptation of appetizing foods in our environment [9] and have poorer weight management [18,19,22].

Encouragingly, there is evidence that using EF training tasks that target impulsivity and attentional bias can shift appetitive behaviors. Inhibitory control is predictive of FV consumption [23], and increased attentional bias toward healthy foods leads to increased consumption of “healthy” snacks (eg, mixed nuts) relative to “unhealthy” snacks (eg, chips) [24]. These findings suggest that an EF training intervention that simultaneously increases inhibitory control while reducing reward and attention responses to nutrient-poor food should improve dietary choices [25-27]. However, to date, there are no examinations of EF training on appetitive behaviors related to increasing FV consumption while concomitantly decreasing the valuation of energy-dense, nutrient-poor foods.

### EF Training

EF training tasks that target attention bias (eg, dot probe) and support inhibitory control (eg, go/no-go) can shift eating behaviors. Training inhibitory control with weekly 30-minute go/no go training sessions for 4 weeks has successfully reduced body weight [28]. Shorter training periods (4 sessions in 1 week) have reduced energy intake and liking of energy-dense foods and promoted weight loss [29]. Overall, the current body of research indicates that EF training can be a key component in improving appetitive behaviors. However, it is unclear whether web-based EF training improves EF as measured via response inhibition and attention or whether EF training can alter attitudes and perceptions of nutrient-poor foods and consumption of those foods while concurrently improving attitudes toward and perceptions and consumption of FV. As such, we have designed a web-based EF training program—EFfect (Executive Function Forming Exercises for Cognitive Training) for food choices, or EFfect-food choices—that is, a suite of 4 games intended to target both attention bias and inhibitory control as they relate to FV consumption.

### Moderators of EF Training

Consumption of complex carbohydrates, omega-3 fatty acids, and protein positively correlate with memory and working memory scores [30]. There is some evidence that greater-than-recommended protein intake is correlated with enhanced EF domains [30]. Conversely, consumption of simple carbohydrates and saturated fatty acids is associated with reduced global cognition and memory and learning scores [30]. This study will test whether habitual dietary intake at baseline moderates the effects of the intervention on baseline EF and with pretest-posttest changes in EF and dietary intake.

Greater time between trainings may contribute to greater reductions in “unhealthy” food intake, potentially because spaced training allows for consolidation of learning, suggesting that the interval between training sessions may be important [29,31]. This study will determine whether gameplay frequencies within weeks and across an 8-week intervention period predicts greater change in primary and secondary outcomes [31].

Chronic stress may also moderate EF training efficacy, although this hypothesis has not yet been tested. Chronic stress is associated with greater difficulties with concentration, memory, and decision-making and with poorer performance on tests of working memory, attention, response inhibition, and cognitive flexibility [32-34]—all constructs of EF. Mechanistically, chronic stress alters brain morphology in areas responsible for EF. Humans who have experienced chronic stress display altered connectivity within frontoparietal networks that mediate attention shifts [35] and atrophy of the medial prefrontal cortex and caudate nucleus [36]. Chronic stress also increases putamen volume and dendritic branching [36]. Thus, chronic stress produces central morphological changes that could reduce EF and increase impulsivity.

Self-reported baseline BMI will be tested as a moderator of the efficacy of the EF training in improving EF scores and reducing BMI. A meta-analysis found that people with obesity display impairment across inhibition, working memory, decision-making, and planning EF domains [37]. However, baseline BMI did not moderate the association between obesity and EF [37]. Excess adiposity may not be a function of overconsumption of energy but, rather, of population-level changes in composition of the gut microbiome, reduced diet-induced thermogenesis in response to consumption of processed foods, the magnitude of browning or beiging of adipocytes, the rate of mitochondrial respiration, and intergenerational transmission of epigenetic alterations that increase the risk of excess adiposity [38]. In addition, given that a period of overeating that results in weight gain is associated with increased reward region responsivity to energy-dense foods [39], it is possible that EF training will be more effective for participants with lower versus higher BMI values.

### Objectives and Hypotheses

The primary objective of this project is to test the efficacy of the EFfect-food choices training program in improving EF and increasing consumption of targeted nutrient-rich FV. Secondary objectives include the effectiveness of the EFfect-food choices training program in (1) increasing an individual’s awareness of dietary choices, (2) increasing positive attitudes toward FV, (3) increasing the valuation and consumption of FV, and (4) decreasing the valuation and consumption of nutrient-poor foods. We will also determine whether changes in self-consciousness of, attitudes toward, and valuation of FV predict change in dietary intake and whether baseline dietary intake, BMI, and psychological stress moderate the efficacy of EF training and food valuation.

The specific hypotheses are as follows: (1) EF training will increase behavioral response inhibition as measured through faster response time, performance accuracy, and delayed discounting; (2) EF training will reduce attentional bias toward nutrient-poor food cues as measured through faster response time and performance accuracy; (3) EF training will reduce intake of nutrient-poor foods in participants who demonstrate greatest inhibitory and attentional learning; (4) time spent playing EF games and number of EF game sessions completed will be positively associated with increases in EF in the EFfect-food choices group; (5) spaced EF training will facilitate greater increases in EF than massed EF training; (6) greater length of delay between the last training day and testing of EF game performance at 8 weeks will be associated with smaller improvements in EF; (7) usual consumption of complex carbohydrates, omega-3 fatty acids, and protein will correlate with baseline EF; (8) usual consumption of simple carbohydrates and saturated fatty acids will be associated with reduced EF; (9) greater baseline and pretest-posttest consumption of complex carbohydrates, omega-3 fatty acids, and protein will correlate with increased EF; and (10) lower usual and pretest-posttest consumption of simple carbohydrates and saturated fatty acids will be associated with increased EF.

## Methods

### Study Design

A single-blinded, parallel, 2-arm randomized controlled web-based clinical trial will be used to test the effectiveness of the EFfect-food choices training program in increasing FV consumption. Only designated study coordinators assigning the study intervention are unblinded. All study procedures are web based and by invitation only. All outcome measures are completed during baseline and at the end of the 8-week treatment period (Table 1). The baseline and posttraining testing periods are 1 week in duration to allow for obtaining 3 days of dietary recall and completion of study questionnaires and EF testing. The intervention consists of 8 weeks of EF training. Details of the EF training intervention are outlined in the following sections. SMS text message or email reminders are sent to participants throughout the study to ensure that all questionnaires and EF testing are completed within 1 week of starting and finishing the EF training intervention and remind participants to complete the EF training intervention each week.

**Table 1:** Testing schedule.

Measurement	Week 0	Weeks 1-8	Week 9
3-d diet records	✓		✓
Questionnaires	✓		✓
EF^a^ testing	✓		✓
EF intervention		✓	

^a^EF: executive function.

### Ethical Considerations

This study has been approved by the University of North Dakota Institutional Review Board (IRB0005432) and is registered on ClinicalTrials.gov (NCT05938894; Train Your Brain—Executive Function; last updated April 7, 2025). Participants are provided with an electronic informed consent form before taking part. No data were collected from participants before obtaining informed consent in accordance with the Helsinki Declaration of 1975 as revised in 1983. After consenting, participants are assigned a study identifier that is used for the collection of all data and randomized to EF training via a suite of 4 web-based games that promote responding to FV, inhibit responses to nutrient-poor foods, and train attention toward the former and away from the latter or sham training that involves images of flowers and songbirds (control). Participants were compensated with US $340 for their time after all study procedures were completed. Any protocol changes will first be approved by the University of North Dakota Institutional Review Board before implementation.

### Recruitment

Participants (N=500 completers) include men and women aged 18 to 70 years who are classified as having a healthy BMI but not greater than the upper end of class II obesity (BMI 18.5-38 kg/m^2^) and are citizens of the United States. Recruitment media may include brochures, newsletters, television, radio, and internet or social media advertisements. Participants are recruited to estimate a nationally representative sample using a quota system based on US census data on sex, age, and race. A reasonable effort is made to achieve these sampling goals, although small deviations may be necessary to complete the study while still meeting the sample size of 500 total completers.

Inclusion criteria include US citizenship, not currently dieting to lose weight and no weight loss or gain of >4.5 kg (10 pounds) over the previous 3 months, no tobacco or e-cigarette use, and not being pregnant or lactating or planning to become pregnant while participating in the study. A history of bariatric surgery; diagnosis of a major medical or psychiatric condition that would interfere with participation, including not being physically able to complete the computer training; or a current eating disorder are additional exclusion criteria.

Potential participants complete an initial prescreen online survey to verify that they meet the study criteria. Eligible applicants are invited to participate in the full study and provided with a web-based informed consent form. After the participant submits the consent form, the information is verified, and a copy of the signed consent form is sent to the email address provided by the participant.

### EF Testing and Training Intervention

#### Overview

EFfect-food choices consists of 4 games designed to test EF as associated with nutrient-rich food (NRF) choices and designed to train participants to search for and shift their attention toward FV, specifically by shifting attentional bias and increasing inhibitory control. The games are constructed to prompt responses to FV 100% of the time and prompt inhibition to nutrient-poor foods 100% of the time in the intervention group. All images are of the same background color (white) and size to allow for consistent alignment within a grid. Participants play each game once before and after the 8-week EFfect-food choices intervention at the same standardized game settings, and the game outcomes, namely, response time, percentage correct, and raw data on errors of omission and commission, will serve as EF outcomes.

For the 8-week EF training intervention, participants play all 4 of the EFfect-food choices games a minimum of twice per week on 2 separate days. A week is defined as beginning on a Wednesday and ending on a Tuesday. Participants who have not yet completed at least 1 of the 2 expected training sessions of each game by Friday at 11:59 PM receive a reminder email or SMS text message to complete the training. Participants are given 1 week to become current with their past-due training, after which they are removed from the study. If necessary, a second email or SMS text message reminder is sent on Monday. Participants can choose the days of the week on which they play the games. Participants have different schedules of daily living, and choice over scheduling of gameplay should help promote adherence. Participants must play the games at least twice per week but can play as many times as they wish. In addition, participants have control over the order in which the games are played. This structure allows for individual differences in gameplay order and frequencies within weeks and across the 8-week intervention period and allows for testing whether the pattern of training correlates with greater efficacy of the intervention on EF. In effect, this strategy allows for testing whether more widely spaced training facilitates greater consolidation of reward learning than massed (ie, consolidated) training.

The following sections provide detailed descriptions of the games for the intervention group.

#### Go/No-Go

**Figure 1:**
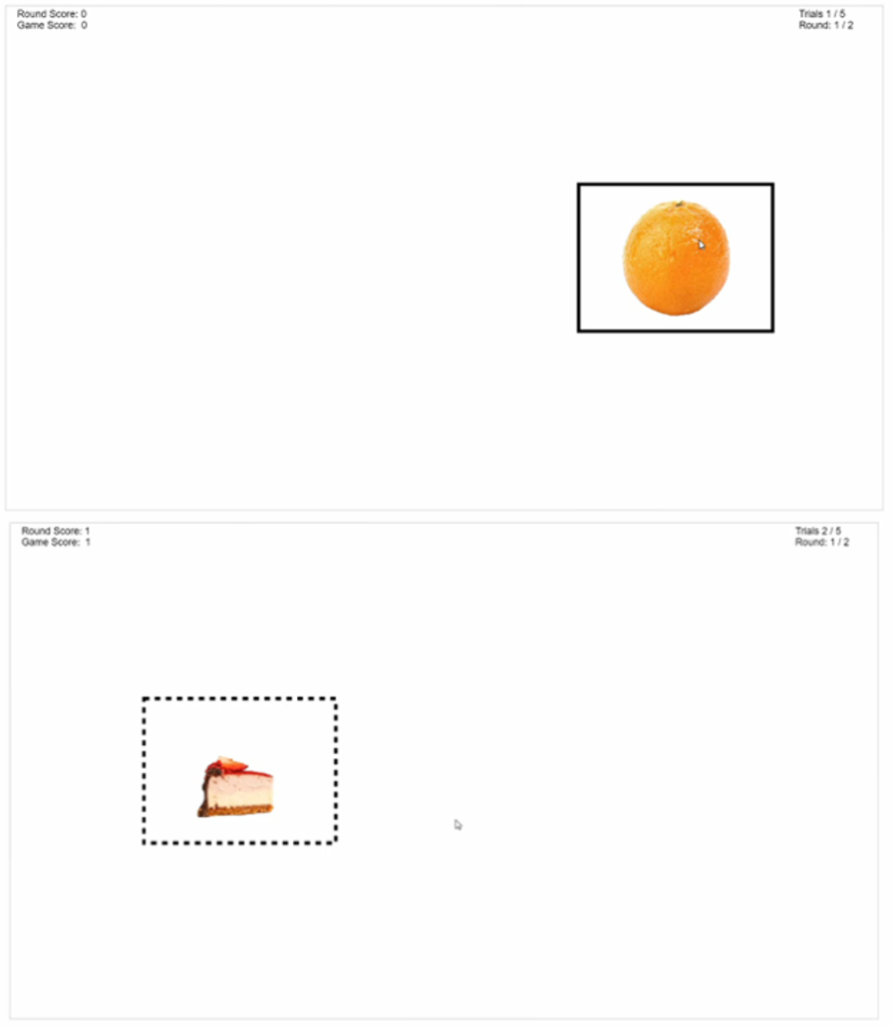
Screenshots of the Go/No-Go game.

Participants are presented with a series of food images appearing on either the left or right side of a rectangular gameplay area that is centered on the screen (Figure 1). Participants are instructed to click on the images that appear in the solid boxes as quickly as possible and not click on the images framed in dashed boxes. For the intervention group, for 100% of the trials, FV images appear in a solid box, and images of nutrient-poor foods appear in a dashed box. If the participant fails to click on an image in a solid box within the allotted time, or if they click on an image in the dashed box, a red *X* is displayed to provide feedback of the error. This trains participants to focus on the FV images when playing the game. Neutral filler images (a water-filled glass) are presented and appear in the solid boxes 50% of the time and in the dashed boxes 50% of the time. The 50:50 ratio of box type for the filler images is to discourage a training effect for these images and permit calculation of inhibitory learning for nutrient-poor foods and FV. A description of the control group sham gameplay is provided in the Control Group section. It takes approximately 10 minutes to complete this game.

#### Stop Signal

**Figure 2:**
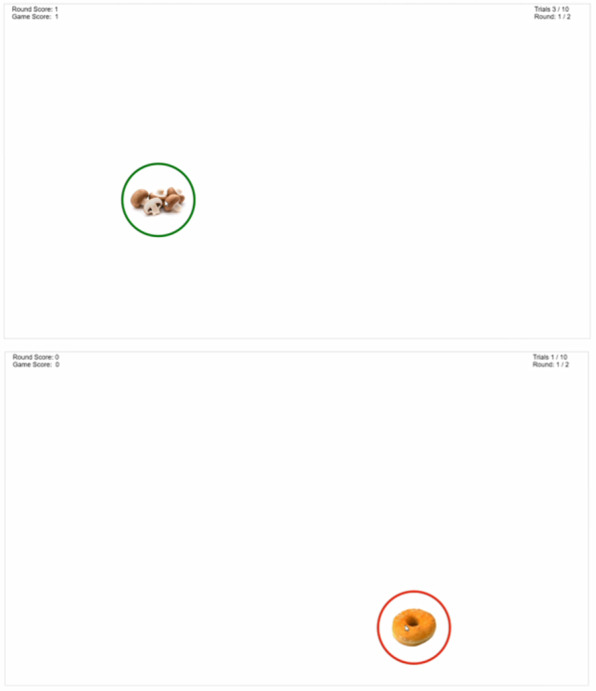
Screenshots of the Stop Signal game.

Participants are presented with FV, nutrient-poor foods, and filler images. These images are surrounded by either a red or green solid border that is delayed in appearance (Figure 2). Participants are instructed to click on the images surrounded by a green border as quickly as possible and not click on images with red borders. Images are presented for a short duration and then removed from the screen. Display duration of the images is discussed in the User Advancement section. When the participant fails to click on the image with a green border in the allotted time or clicks on the image that has a red border, a red *X* is shown on the screen. The FV images and nutrient-poor foods are framed with the solid green border 100% and 0% of the time, respectively. The filler water images are framed by the solid green border 50% of the time. It takes approximately 8 minutes to complete this game.

#### Dot Probe

**Figure 3:**
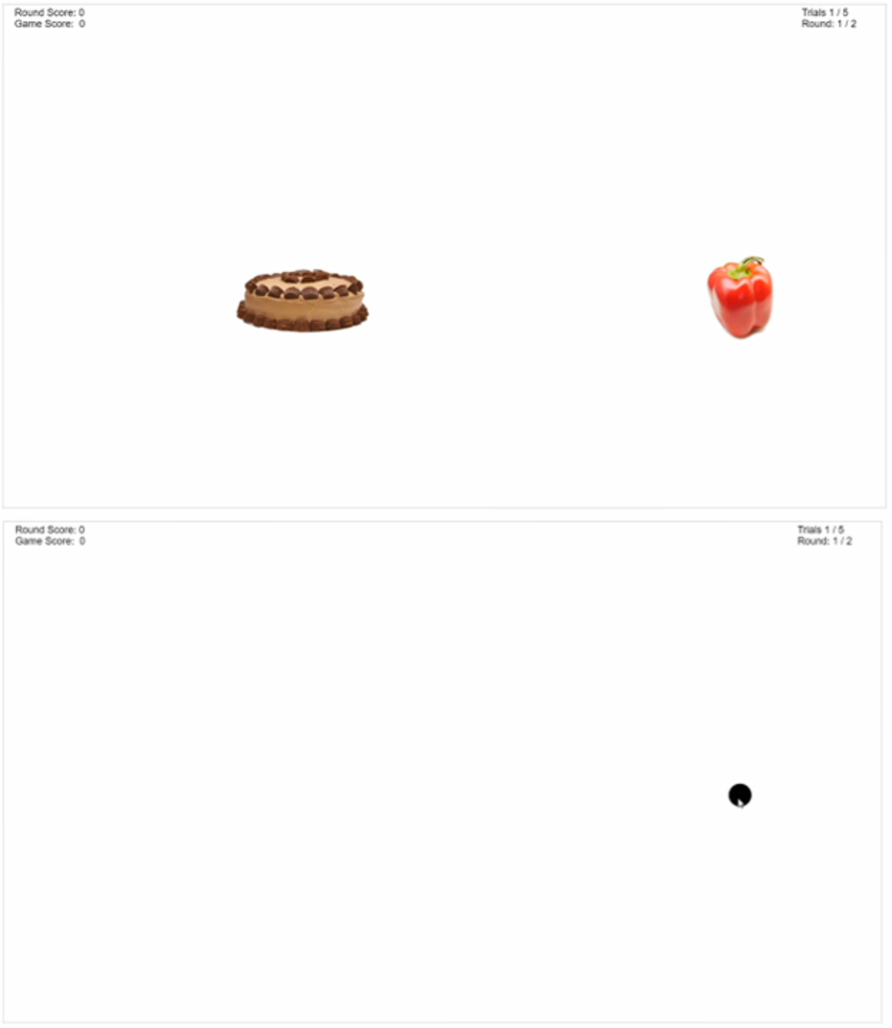
Screenshots of the Dot Probe game.

Pairs of a randomly selected FV image and a randomly selected nutrient-poor food image are shown on the screen (Figure 3). The images are shown side by side for a set duration (see the User Advancement section), and each individual image of the pair is shown on both sides of the rectangle gameplay area an equal number of times. Before the image pair is shown, a fixation cross appears in the center of the gameplay area for 500 ms. After the image pair disappears from the screen, a small black dot appears in the area that one of the images in the pair occupied and remains visible until a response in the form of a mouse click is registered in the gameplay area. Unlike the go/no-go game, a red *X* does not appear if the user does not click on the dot. Participants are instructed to click on the dot as fast as possible once it appears. In 100% of the trials, the dot appears within the space that the FV image occupied. The location of the dot changes for each image pair to prevent the participant from maintaining a single mouse position for the entire game. It takes approximately 14 minutes to complete this game.

#### Visual Search

**Figure 4:**
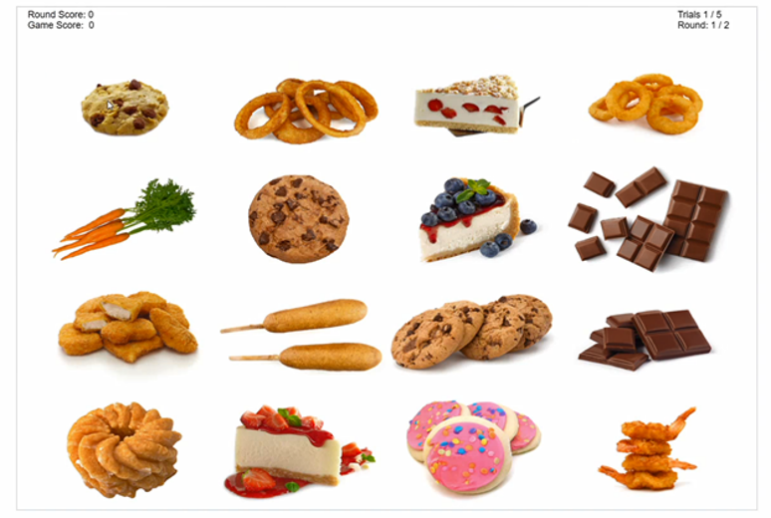
Screenshot of the Visual Search game.

Participants view a 4 × 4 array of randomly selected food images and then search and click on the FV image as quickly as possible within the allotted time (Figure 4). Each array consists of 1 randomly selected FV image and 15 randomly selected images of nutrient-poor foods (Figure 4). When the participant successfully clicks on the target FV image, the other 15 images disappear, and the target FV image is centered and framed with a green rectangle and becomes larger in size until the next set of images appears. If the participant fails to find the target FV image or clicks on one of the nutrient-poor images in the array, the images are removed from the screen, and a red *X* appears. It takes approximately 6 minutes to complete this game.

### Image Selection

Image selection for the games was strategic and based on nutrient density as determined using the NRF Index (the Nutrient Rich Foods Index). The NRF Index is a method of quantifying nutrient density that has been validated against the Healthy Eating Index score [40]. The NRF 9.3, a scoring system within the NRF family of indexes, provides a numerical score for individual foods that is calculated by subtracting the sum of daily values of 3 nutrients to limit from the sum of daily values of 9 nutrients to encourage (thus, 9.3) per 100 kcal [41]. The 9 nutrients to encourage on the NRF 9.3 scale are protein, dietary fiber, vitamin A, vitamin C, vitamin E, calcium, iron, potassium, and magnesium, and the 3 nutrients to limit are added sugar, saturated fat, and sodium. For the purposes of calculating NRF Index values for this study, total sugar was used in place of added sugar due to data availability in the Food and Nutrient Database for Dietary Studies. The NRF Index score does not provide cutoffs for levels of nutrient density (ie, nutrient dense vs nutrient poor). For this study, nutrient-poor foods were defined as those foods with an NRF Index score of ≤15.

Images were purchased through Shutterstock (Shutterstock, Inc), and all images feature a single food item with a blank, white background. A total of 80 FV images (40 of fruits and 40 of vegetables) and 80 nutrient-poor food images, which include both sweet (ie, candy, cake, and ice cream) and savory (ie, fried chicken, onion rings, and pizza) foods were selected. The FV images include 9 fruits (pineapple, kiwi, peach, orange, berries, melon, grapes, banana, and apple) and 14 vegetables (asparagus, broccoli, Brussels sprouts, carrots, cauliflower, celery, corn, cucumber, lettuce, mushrooms, green peas, sweet peppers, radish, and tomato). The average NRF Index for all images is 83.7 (SD 132.4). The average NRF Index score for the FV depicted in the images is 169.2 (SD 142.8). A total of 13 types of nutrient-poor foods were selected, including cake, candy, cheesecake, chicken nuggets, cookies, corndogs, cupcakes, doughnuts, fried chicken, ice cream, onion rings, pizza, and milkshakes. The average NRF Index score for the nutrient-poor foods depicted in the images is −1.79 (SD 7.77). In addition to these images, 40 images of glasses of water were selected for use as neutral filler images. The filler images are paired with inhibition cues and attention probes on a 50:50 ratio and allow for calculating inhibition and attentional learning for nutrient-poor foods and FV images.

### Control Group

**Figure 5:**
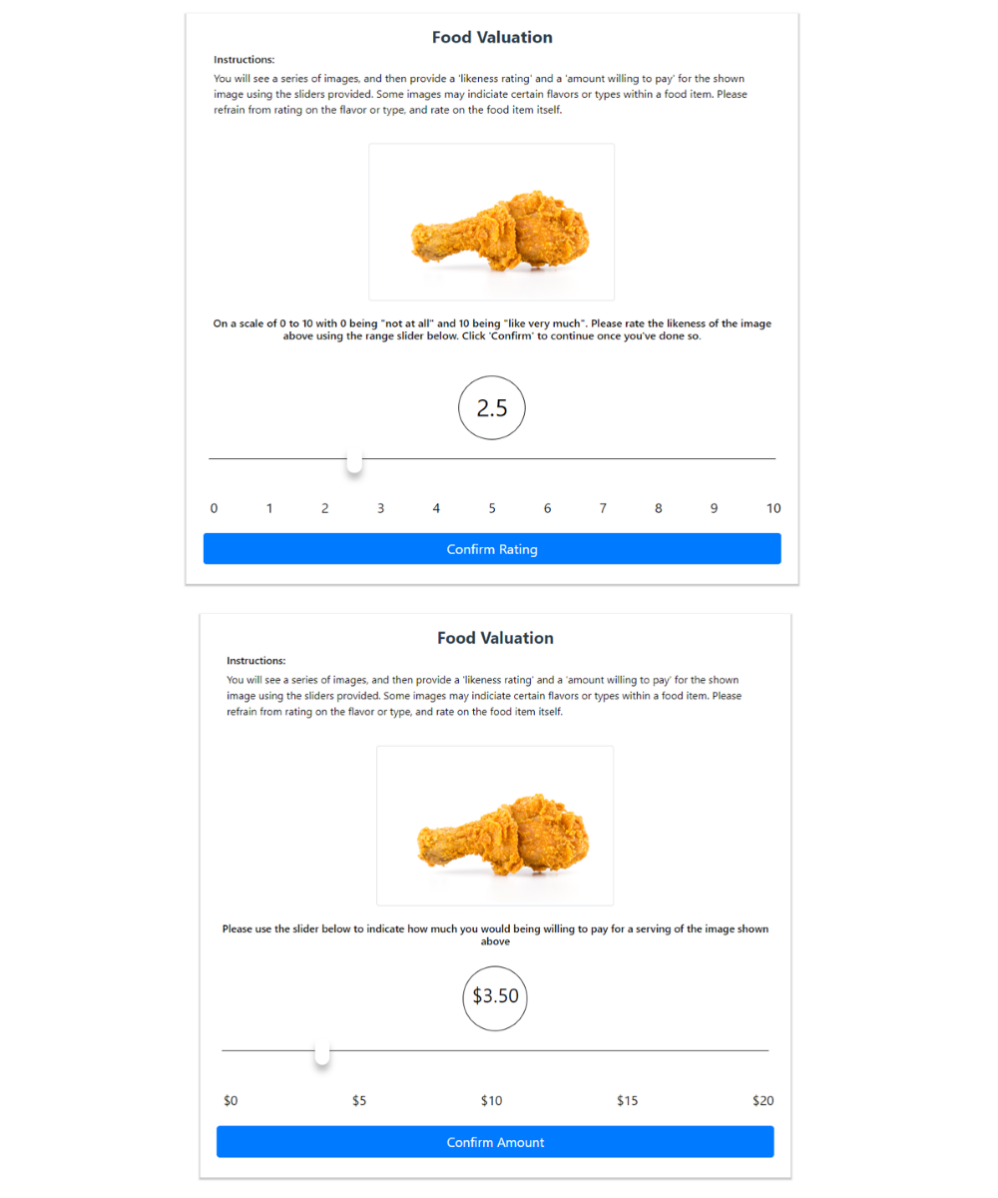
Screenshots of the Willingness to Pay assessment.

Participants randomized to the control group play nearly identical versions of the games except that, in place of foods, they are presented with images of flowers and songbirds. The control group is randomized at a ratio of 1:1 to have either flowers or songbirds as the images used in place of FV. For participants randomized to have flowers in place of the FV images, images of songbirds are used in place of nutrient-poor foods (Figure 5). For participants randomized to have songbirds in place of the FV images, images of flowers are used in place of nutrient-poor foods. Participants are informed through the study material that this training improves inhibitory and attentional control, which should reduce the risk of overeating. Similar to the intervention condition, images were selected and purchased from Shutterstock and have a plain, white background featuring only 1 songbird or flower. A total of 80 flower and 80 songbird images on a blank, white background were selected, and the images were evenly distributed to not overrepresent a given species. Participants in the control group are also required to play each game twice per week on separate days. All control participants complete the same series of measures and tests as those in the intervention group.

### Treatment Fidelity and Training Volume

Treatment fidelity is encouraged through gamification strategies and reminder messages. Treatment fidelity and training time are measured using the web-based EFfect-food choices program as it provides a record of when and how often each of the games is played. This record provides real-time data regarding use of the EFfect-food choices program. Treatment fidelity and training volume measures include total time spent playing EFfect-food choices games, total number of sessions completing all 4 EFfect-food choices games, and total number of any of the EFfect-food choices games completed.

### Gamification Strategies

To test whether EFfect-food choices with its fully online platform is more scalable and translatable than other EF training programs, participants complete all EF training outside a laboratory setting. To improve gameplay adherence, gamification theory [42-44] was applied to the development of EFfect-food choices. Gamification is commonly used in health-related apps, especially those targeting physical activity and weight loss [44]. Gamification includes features to increase engagement with nongame programs, such as badges, leaderboards, challenges, and adaptive features that are more effective (relative to games without adaptive features) in increasing EF [42]. A review of gamification suggests that, overall, gamification enhances engagement with health games; increases motivation, enjoyment, and understanding; reinforces the behavior; and increases adherence [45]. This strategy should increase participant motivation to continue playing the games and, thus, improve the efficacy of this trial as participants continue to engage with the treatment. If found to be effective, the impact of this research would be a low-cost, practical, easily accessible form of EF training that simultaneously increases FV intake while reducing consumption of nutrient-poor foods and that can be widely distributed to the general population via PC, tablet, or smartphone. Using the taxonomy of gamification approaches of Schmidt-Kraepelin et al [46], the gamification concepts of the EFfect-food choices training program are identified and classified (Textbox 1).

**EFfect-food choices training program gamification concepts.**

1. Gamification messages are directly conveyed to users via text outputs.
2. User identity is determined from the user’s first name and the first initial of their last name. The user’s identity is displayed as a generic user icon on the user’s account page.
3. Rewards or reinforcement are accessible only internally to the game. Badges are specific to each game to promote playing of all games. Earned badges are retained for all 8 weeks. Badges are unlocked when achieving a score within the top 10 on any game leaderboard for the current week or month or on the all-time leaderboard; achieving 100% correct answers in all trials on the easiest, normal, and hardest difficulty levels; the user plays the game for the first time; and the user plays the game for the 10th time. Positive messages are used to encourage gameplay. Participants receive 1 of 20 positive messages at the end of each round of a game and the end of each game. Participants also receive positive messages when achieving the milestones presented in Table 2.
4. Competition is in the form of comparison of gameplay to oneself and with all other users via point systems and leaderboards. Leaderboards are categorized as weekly, monthly, and for all time. Scores on the weekly leaderboard are reset at the start of the week, and scores on the monthly leaderboard are reset on the first day of each month to motivate continued gameplay across all 8 weeks.
5. The target group is individuals with a normal weight to obese but otherwise healthy who are seeking to eat healthier.
6. There is no collaboration or goal setting.
7. Regarding narrative, the gamification concept is reset to the starting difficulty at the beginning of each round of new play. Nothing persists from one gameplay to the next. The games adjust in difficulty between each round of the game session depending on performance.
8. Regarding level of integration, the gamification concepts are, for the most part, independent as gameplay could occur without the additional gamification elements. Gamification elements have been introduced to promote gameplay.
9. Regarding user advancement, an adaptive feature increases the difficulty of gameplay based on accuracy and speed of performance. The difficulty and speed level ranges from 0 to 10, with 0 being the easiest or slowest and 10 being the hardest or fastest, with the default difficulty level being 5. Scoring >90% accuracy prompts the game to increase in difficulty, and scoring <70% accuracy prompts the game to decrease in difficulty.

**Table 2:** Positive messages for attaining milestones.

Milestone	Message
Weekly high score badge	“That is your weekly high score! You’re getting better every day.”
Monthly high score badge	“Best score yet this month!”
All-time high score badge	“All-time high score! You did very well.”
Easy ace (easily completes the low-difficulty level)	“You’re learning fast.”
Normal ace (easily completes the difficulty level 5)	“What an improvement! You’ve got it now.”
Hard ace (easily completes the high-difficulty level)	“Great improvement! You just aced a hard difficulty level.”
Playing the game for the first time	“Thanks for playing the game for the first time. Nice going!”
Playing the game 10 times	“That’s the 10th time you’ve played the game. Keep up the great work!”

### Gameplay Pattern

The massed-spaced training effect (playing all the games in a single session compared to playing the games across several days) measures include (1) the mean and SD of the number of days between training sessions; (2) the mode of the number of days between training sessions; and (3) the density index, which is calculated using the model established by Aulbach et al [47], where the number of trials completed on the most active day is divided by the total number of trials. For the first 2 indexes, a greater value indicates greater spacing of training. For the density index, a greater value indicates greater massing of training.

### Questionnaires

Two questionnaires are completed once at the beginning of the study. First, in the demographics questionnaire, participants are asked to report their age, sex, gender identity, ethnicity, weight, height, household income, educational level, marital status, employment status, income (individual and household), and food insecurity. Food insecurity is assessed using the Six-Item Short Form of the Food Security Survey Module [48].

Second, the Weight History Questionnaire assesses self-reported weight and was revised from the Weight History Questionnaire used for the National Health and Nutrition Examination Survey 2011 to 2012. The questionnaire also asks participants to report perceptions of their own weight, such as whether they believe they are over- or underweight or if they want to gain, lose, or maintain their weight. Participants also report their history with weight management, such as their highest past weight and whether they have attempted to lose weight and, if so, how.

There are also several questionnaires that are completed before and after the 8-week EF training intervention. The Automated Self-Administered 24-Hour Dietary Assessment Tool is used to measure food and drink consumption [49]. Participants complete three 24-hour dietary recalls before starting the intervention and 3 upon completion of the intervention. To lessen the day-of-the-week effect, 24-hour dietary recalls are randomly assigned so that 2 are collected on a weekday (Monday-Thursday) and 1 is collected on the weekend (Friday-Sunday). Participants report all meals, snacks, and drinks consumed from lists provided and may add anything they consumed that is not on the list. The number of FV cup equivalents consumed and Healthy Eating Index scores are calculated for each day that participants report their dietary intake to determine the efficacy of the EFfect-food choices training program in altering dietary intake patterns.

The Three-Factor Eating Questionnaire–Revised-18 is used as a measure of how people interact with food [50]. The 3 factors measured include cognitive restraint (eg, “I deliberately take small helpings as means of controlling my weight”), uncontrolled eating (eg, “Sometimes when I start eating, I just can’t seem to stop”), and emotional eating (eg, “When I feel anxious, I find myself eating”). All items are rated on a 4-point scale. Mean scores are calculated for each category.

The Power of Food Scale measures appetitive responsiveness to the rewarding properties of the food environment [51]. The Power of Food Scale is a 15-item questionnaire with a 5-point Likert-type scale ranging from 1 (do not agree at all) to 5 (strongly agree). Items are scored so that a greater score indicates greater responsiveness to the food environment.

The Food Attitudes and Behaviors survey is used to explore potential factors associated with the incorporation of FV into the diet [52]. The Food Attitudes and Behaviors survey measures domains of social support, perceived barriers, perceived benefits, motivation, and self-efficacy of FV consumption.

The transtheoretical model is a theory that posits that people’s behavior change can be classified into 1 of 6 stages, ranging from no intent to change to the change being made and maintained [53]. One scale that assesses this is the Health Behavior and Stages of Change Questionnaire [54]. This scale asks participants to select the stage that represents their stage of change for a variety of health practices, such as physical activity and seeing a nutritionist. For this study, we ask participants to complete the nutrition questions from this scale, which includes modified questions to reflect increasing consumption of FV and decreasing consumption of nutrient-poor foods.

Participants’ health consciousness is measured using a brief 11-item scale [55]. Participants rate the extent to which they agree with statements such as “I’m very self-conscious about my health” and “My health depends on how well I take care of myself.” This measure uses a 7-point Likert-type scale that ranges from 1 (strongly disagree) to 7 (strongly agree) [55].

Self-efficacy, or the belief in one’s own ability to perform a behavior successfully, is measured via the New General Self-Efficacy Scale [56]. This is an 8-item measure that uses a Likert-type scale ranging from 1 (strongly disagree) to 5 (strongly agree).

To ascertain whether “usual stress” impacts the efficacy of EFfect-food choices in increasing EF, participants are asked to complete the Perceived Stress Scale [57]. The Perceived Stress Scale is a brief, 10-item questionnaire that asks participants to indicate the frequency with which they have experienced a range of stress-related emotions during the previous month. This measure uses a 5-point Likert-type scale ranging from 0 (never) to 4 (very often).

Chronic stress is assessed via the English version of the Trier Inventory for Chronic Stress [58] that retains 9 factors derived from 57 items about situational experiences during the previous 3 months. The inventory’s response options are never, rarely, sometimes, frequently, or very often. The short form correlates (*r*=0.94) with the original Trier inventory [58].

Similar to the methods by Smith et al [59], at each EF testing session, participants’ level of hunger, fullness, thirst, and boredom are measured using a separate on-screen slider cross-modal visual analogue scale (VAS; range from 0 to 10 at 0.1-unit increments) and anchored by 0 (not at all) and 10 (extremely). Desire to eat is assessed using a VAS anchored by 0 (none) and 10 (maximum desire) [59].

Valuation of nutrient-rich and nutrient-poor foods was adapted from the methods by Stice et al [26]. A standard image for each of the 9 fruit types, 13 vegetable types, and 14 nutrient-poor foods was chosen by the investigators to establish a standard deck of 36 assessment images that most closely approximate a similar portion size. The deck of images is randomly sorted into a presentation order that becomes the standard order of presentation for all participants on each test date. Figure 5 shows an example of how liking and valuation are presented to the participants. Valuation of the 36 foods appearing in the images is conducted after at least 2 hours of not eating, in a single session, and one food at a time. With the image of the single food displayed on the screen, participants then use an on-screen slider cross-modal VAS to provide their liking (range from 0 to 10 at 0.1-unit increments) of each of the nutrient-rich and nutrient-poor foods. The VAS is anchored by 0 (not at all) and 10 (like very much). After clicking on a submit button, the liking scale disappears. A second VAS appears, and participants assess how much they would be willing to pay (range from US $0 to US $20 at US $0.25 increments) for the displayed amount of each of the foods, which have been selected to correspond to commonly consumed serving sizes. Upon clicking on the submit button, the next food appears, and the process is repeated.

The 27-item monetary choice questionnaire by Kirby et al [60] asks the participants to make a choice between a smaller, immediate monetary amount (S) and a larger, delayed monetary amount (L). For example, participants are asked the following: “Would you prefer (a) $19 today or (b) $25 in 53 days?” The 27 items are grouped into 3 categories based on the value of the rewards: small (US $25-$35), medium (US $50-$60), and large (US $75-$85). Discount rates are calculated separately for each category using the syntax by Gray et al [61] to determine whether the size of the reward affects discount rates.

In addition, the System Usability Scale (SUS) is completed once at the end of the study. The SUS is used for online usability surveys and provides a global measure of system satisfaction [62]. The SUS uses a 5-point scale with anchors of 1 (strongly agree) and 5 (strongly disagree). Participants complete the SUS with minor revisions to the wording of several items to make them easier to comprehend [63]. A 1-item adjective scale is added to understand the usability associated with an individual’s global SUS score [63].

### Evaluation Outcomes

Improvements in response time and percentage correct are indicative of improved EF. EF testing is completed by playing the EFfect-food choices games at the initial (default) speed at both baseline and postintervention testing. Participants complete the EF testing on the same device type (ie, desktop, laptop, or tablet) at baseline and after completing the intervention. Study personnel remind the participants to complete the games on the same device type. The game records the type of device used to play. Records are checked to assess whether the games were played on the same device type at each testing session.

### Data Validity

Following the recommendations of Jones et al [64], filtering methods to improve the validity of the data include low-probability (LP) screening questions presented near the beginning of the assessment questionnaire and trap questions placed immediately before the most critical sections of the questionnaire measures. At the beginning of the survey, LP screening questions are used to identify falsely answered screening questions to attempt to qualify for the study and the use of software to auto-complete surveys. In addition to purchasing fruits or vegetables in the previous year (study inclusion criterion), participants are asked to select specific fruits (fresh bananas, strawberries, grapes, apples, watermelon, oranges, blueberries, and peaches) and vegetables (fresh broccoli, cabbage, carrots, cucumbers, peas, peppers, potatoes, and tomatoes) that they have purchased in the previous year. Respondents could also select LP fresh options of fresh mangosteen, red currants, and romanesco broccoli. The very limited availability of these options makes it unlikely that a participant will have purchased 2 of the 3 LP fresh options in the previous year. Per Jones et al [64], “medium” and “high” gaming of the system occur when participants select 2 or all 3 LP options, respectively. A trap question is inserted just before the primary outcome sections of the questionnaire. The trap questions are modeled after the work by Jones et al [64]. The available response options for the trap questions are strongly disagree, disagree, agree, and strongly agree.

### Data Quality

The quarantine strategy by Jones et al [64] is used to enhance data quality by flagging those responses for which there has been medium or high gaming via the LP questions and additional failure of a trap question immediately before each questionnaire section. If the aforementioned is true, the data for the sections that do not have a preceding trap question will also be evaluated for threats to validity with quarantining of those sections occurring due to either straight-lining (>40% of all questionnaire sections completed with response nondifferentiation) or “speeding” (mean response completion times for the section of <4 seconds per question). Descriptive statistics will then be determined, and the distribution of each variable will be examined to assess normality. Appropriate transformations based on the distribution will be considered before analysis, as will the assessment of outliers and conduct of sensitivity analyses in which they are omitted or winsorized. Winsorizing is a method of averaging that limits the effect of extreme values and reduces the effect of possibly spurious outliers [65].

### Statistical Analysis

#### Power

On the basis of means and SDs from go/no-go and dot probe task reaction time results by Stice et al [26], the desired power of 0.9 is reached for the pairwise comparison of the control and intervention groups at week 8 for go/no-go and dot probe task response time with 448 participants and 532 participants, respectively. Multiple comparisons used for the power calculations were the Tukey honestly significant difference tests. On the basis of power calculations, data collection will continue up to the collection of complete data from 500 participants. On the basis of the work by Jones et al [64], we expect the data of approximately 15% of participants completing a single-setting online questionnaire to be quarantined. Attrition is also likely across an 8-week study, which we estimate will be another 5% of the participants.

#### Data Analysis

Analyses will be conducted using SAS (SAS Institute), SPSS (IBM Corp), and Systat (Grafiti LLC). The analytic plan to determine whether the EFfect-food choices training program improves EF as measured via response inhibition and attention bias is to use linear mixed-effects models to assess group changes in EF outcomes from each of the 4 EF games over time. Sex, race, and age will be tested as covariates in models of all primary and secondary outcomes. For each EF outcome variable, the mean scores from the first 3 to 5 sessions at baseline and the mean scores of the last 3 to 5 sessions completed at 8 weeks will be used as data.

Group and sex differences in rates and proportions of participants violating measures of poor response behavior and of data being quarantined will be assessed using log-linear models. Per the CONSORT (Consolidated Standards of Reporting Trials) guidelines [66], testing of group baseline differences will be based on the “prognostic strength of the variables measured and the size of any chance imbalances that have occurred.” Group baseline differences will be tested using statistics appropriate for the level of measurement of the data. Demographic differences between quarantined and nonquarantined participants will also be tested using linear regression or mixed models.

The analytic plan to test the independent and interactive associations between stronger inhibitory and attentional learning and greater reductions in intake of nutrient-poor foods is to use mixed model regression. Variables that are correlated to change in consumption of nutrient-poor foods will be tested as covariates in these mixed models. Establishing dose-response relationships is an important element of establishing the efficacy of an intervention. As such, training time as measured through total time spent playing EFfect-food choices games, total number of sessions completing all 4 EFfect-food choices games, and total number of any of the EFfect-food choices games completed will be tested as predictors of outcomes in the intervention condition.

The analytic plan to determine whether the EFfect-food choices training program improves awareness of dietary choices, positive attitudes toward FV, valuation of FV, and consumption of FV and lowers consumption of nutrient-poor foods and body weight is to use separate mixed models to test differences between groups (control and intervention) and across time (baseline and 8 weeks). EF treatment fidelity and any significant between-group differences in variables that are correlated with outcomes will be tested as covariates in these mixed models. Body weight response data will be tested in all participants and in the subset of participants with a BMI of >25 kg/m^2^.

For all models, any significant between-group differences in variables that are correlated with outcomes will be included as covariates in the mixed models. Outcomes will be tested with an intention-to-treat approach using (restricted) maximum likelihood estimation [67] where all participants who are randomized will be included in the data analysis. This approach is associated with more accurate parameter estimates than listwise deletion or last observation carried forward approaches and is appropriate when missingness is correlated with study variables. We will also conduct a completer analysis that includes only those participants who completed the training at least twice per week and the pre- and posttesting.

## Results

### Participants

Recruitment began in September 2023 and is expected to continue until December 2027 or January 2028. As of June 1, 2025, a total of 899 individuals have applied to participate in the study. Of these 899 applicants, 763 (84.9%) qualified and 630 (70.1%) consented. There have been 386 withdrawals due to noncompliance with study requirements (n=342, 88.6%) or being identified as bots (n=35, 9.1%). A large proportion of those withdrawals (141/386, 36.5%) were due to not completing the preintervention questionnaires. To date, a total of 199 participants have completed all study requirements, with 17 (8.5%) in active gameplay and 27 (13.6%) in postintervention testing.

### Study Outcomes

Due to blinding, all data analysis will begin after the last participant has completed all study requirements (anticipated start in January 2028 or February 2028). Results are expected to be published in 2028.

## Discussion

### Expected Findings

The Dietary Guidelines for Americans recommend regular consumption of FV as part of a healthy dietary pattern [68], yet consumption continues to be below the recommended amounts set forth by these guidelines across all age groups, with few exceptions [68,69]. Much research has been aimed at trying to increase FV consumption, with limited success. Intervention studies that provide participants with an allotment of FV demonstrate that FV consumption can be increased [70-72]. However, after the intervention is completed and FV are no longer provided, most return to habitual levels of consumption. This is likely because incentive sensitization does not readily occur with repeated vegetable consumption in adults [72]. Therefore, interventions that target the decision-making process may produce longer-lasting results.

A driver of healthy food choice is executive control in environments in which nutrient-poor foods are widely available and heavily advertised. Research suggests that EF training can help improve self-regulation, decision-making, and impulse control, potentially leading to healthier eating habits [28,29,73,74]. As such, EF training is emerging as a promising new method to change eating behaviors that provides a distinct advantage over other, more traditional interventions [75-78]. EF training may lead to more intrinsic motivation, strengthening the capacity to form and maintain healthy habits, resulting in more enduring behavior changes due to enhanced self-regulation capabilities.

To date, there are no examinations of longer-term cognitive training on eating behaviors. This project will fill this gap and be the first to use EF training targeting inhibitory control and attention bias as an intervention tool to increase valuation and consumption of FV. We expect that the EFfect-food choices training program will increase participants’ ability to reduce their selection of nutrient-poor food options and redirect their attention to FV.

### Potential Impact and Significance

The broader public health impact of the proposed EFfect-food choices program could be significant. By enhancing EF, individuals may become better equipped to make healthier food choices, especially in the context of FV consumption. This may lead to the development of healthier eating habits that persist over time, thereby creating a “culture of healthy dietary choices.” As participants engage in healthier eating behaviors, they might experience improvements in mood, anxiety, and overall mental wellness. The online format of the program allows for widespread accessibility, possibly reaching underserved populations who may have limited access to traditional health interventions. This can help bridge health disparities among different demographic groups. The ability to change the images means that the platform can be implemented among populations with limited access to fresh produce by using images of the types of FV that are locally available.

### Limitations

This study is entirely web based, which is a key strength but also the primary limitation. This study provides a unique opportunity to conduct a randomized controlled trial in a nationally representative group and decreases the potential of observer bias (Hawthorne effect) [79]. The key limitation to conducting this web-based study is ensuring compliance with study requirements. However, the EFfect-food choices program records gameplay and generates a weekly report that can be used to send reminder emails to stay on track with gameplay requirements. Another potential weakness is that the pre- and posttesting may not be conducted on the same device type (ie, desktop, laptop, or tablet), operating system, or browser, which could add variability to the response data. Bridges et al [80] found that, when removing the intertrial variability in response times due to a human user, reaction times varied across browsers but precision within a software package, operating system, and browser combination was small and within the range of 0.2 to 5.0 ms. This study was powered on a between-group reaction time difference during the go/no go task of 33 ms, much greater than the variability reported by Bridges et al [80]. Initial instruction and the reminders provided before each testing session should increase adherence to using the same device and browser, although this cannot be strictly enforced. The device type used by each participant for each testing session is recorded and will be tested as a covariate in statistical models. The reaction time data will be averaged across trials, which should minimize the effect of variability in response times due to the platform, operating system, browser, and device combinations on statistical results [80]. Another potential limitation is that the use of weight control medications is not considered as an exclusion criterion. Weight loss or gain of >4.5 kg over the previous 3 months is an inclusion criterion, and enrolled participants will complete a weight history questionnaire that gathers data on weight control attempts and how the participant tried to change their weight. In addition, response bias (ie, social desirability and demand characteristics) may limit the reliability of the findings. The large sample size and the anonymity that comes from conducting a study completely online will minimize participant bias and increase the accuracy of the results. It is also well known that self-reported dietary intake can be inaccurate, although the fact that we are using a placebo control condition should result in similar bias in both conditions. Finally, our training delivery, individual differences, engagement, contextual factors, and cognitive load may limit the impact of EF training on our outcome measures. The questionnaires that participants complete immediately before and after the EF training will provide an insight into limitations and allow us to determine how these moderate the effect of the EF training.

### Conclusions

The relationship between EF and healthy behaviors may be key to improving diet quality given the obesogenic environments that many Americans are exposed to everyday. If shown to be effective, the EFfect-food choices program will provide a platform that can be used to increase FV consumption and, thus, improve diet quality. The capability of this EF training program to be broadly implemented via the internet will greatly expand the reach of interventional studies designed to aid individuals in food-related decision-making. The program allows for weekly planned training and timely training before anticipated exposures to environments that may require enhanced executive control to promote healthier food choices. This type of intervention can readily be made available to the public.

## Abbreviations

CONSORT: Consolidated Standards of Reporting Trials
EF: executive function
FV: fruits and vegetables
LP: low-probability
NRF: nutrient-rich food
SUS: System Usability Scale
VAS: visual analogue scale

## Data Availability

The data, codebook, and analytic code will be made available from the corresponding author on reasonable request after the study has concluded and all manuscripts have been published.
